# Optimization of a Rolling Triboelectric Nanogenerator Based on the Nano–Micro Structure for Ocean Environmental Monitoring

**DOI:** 10.1021/acsomega.1c02709

**Published:** 2021-08-02

**Authors:** Huamin Chen, Jun Wang, Aifeng Ning

**Affiliations:** † Donghai Institute of Ningbo University, Ningbo University, Ningbo City, Zhejiang Province 315211, China; ‡ Fujian Key Laboratory of Functional Marine Sensing Materials, Center for Advanced Marine Materials and Smart Sensors, Minjiang University, Fuzhou City, Fujian Province 350108, China

## Abstract

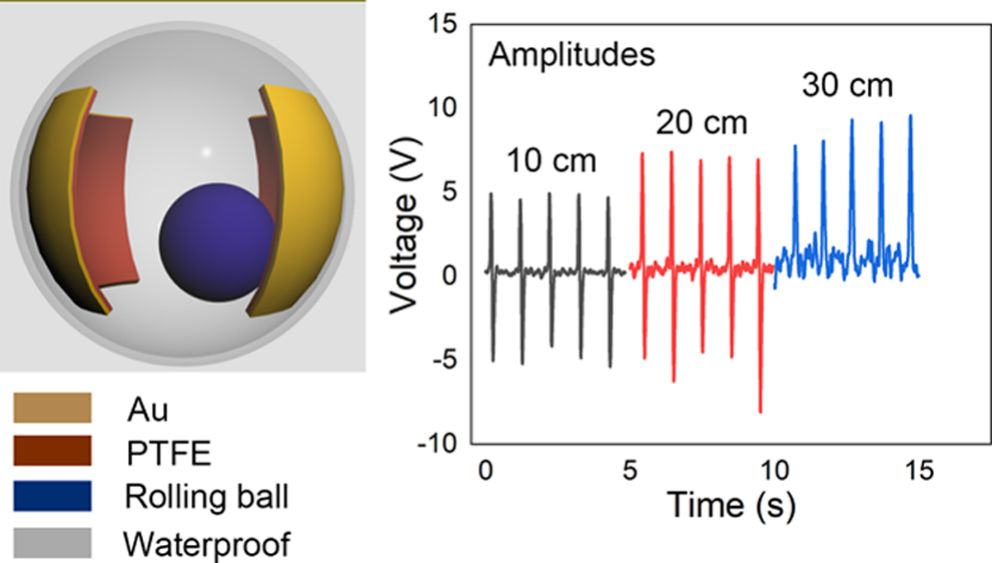

The serious environmental pollution and energy crisis have become a global issue, which makes it a pressing task to develop sustainable and clean energy sources. There exists a large amount of renewable energy in the ocean; unfortunately, most resources are underutilized. In this work, we demonstrate a performance-enhancing rolling triboelectric nanogenerator (TENG) based on nano–micro-structured polytetrafluoroethylene (PTFE) films. The nano–micro structure on the PTFE surface can increase the effective contact area and enhance the triboelectric effect, which is beneficial to improve the output performance. As a result, the output voltage and output current are 25.1 V and 7.3 μA, respectively. We further investigate the effect of nano–micro PTFE concentration on the output performance. The TENG based on a 45% concentration of nano–micro PTFE presents the maximum output power. Furthermore, this TENG can effectively harvest water wave energy with various amplitudes and frequencies, which has the potential to harvest ocean energy for environmental monitoring.

## 1 Introduction

With the increase of environmental pollution issues and energy crisis, the research on renewable and sustainable energy source has been drawn much attention recently.^1−6^ There exist abundant water resources in nature, which contain tremendous green energy.^7,8^ Unfortunately, the extensive ocean energy is underutilized nowadays, which is primarily harvested by the magnetic generator technology.^9,10^ The energy harvesting devices based on the magnetic generators are usually made of complex metal components.^11^ The exposure of these components to seawater will cause pollution to the environment and performance degradation of the devices. More importantly, the miniaturization of the magnetic generator is difficult, and its efficiency is decreased under random and low-frequency seawater.^12^

Recently, a triboelectric nanogenerator (TENG) originated from Maxwell’s displacement current^13−15^ has been identified as a potential candidate for harvesting ocean energy due to its outstanding characteristics, including a simple fabrication process, high cost and performance ratio, high efficiency, and multi-functionality.^16−23^ Great achievements have been made with many prototypes, which are based on the liquid–solid friction mode,^24−27^ solid–solid friction mode,^28−33^ and hybrid mode.^34−37^ In addition, the performance comparisons of different TENG-based water wave harvesting technologies are listed in Table S1. Nevertheless, it is obvious that the performance of TENG would be largely decreased when one of the friction materials is exposed to seawater.^38^ Therefore, a waterproof layer is usually adopted to protect the device from the environmental atmosphere. The rolling spherical structure is an effective and simple design to harvest low-frequency seawater energy without significant environmental pollution and performance degradation of the devices. However, the structure and material parameters of the rolling ball are still needed to be optimized to improve its output performance for practical applications.

In this work, we demonstrate a performance-enhancing TENG based on polytetrafluoroethylene (PTFE) films with a nanostructured surface for seawater energy harvesting. By introducing the nano–micro structure onto the surface of the PTFE film, the effective contact area and friction effect are largely enhanced. As a result, the output voltage and output current are 25.1 V and 7.3 μA, which are increased about 8 times and 2 times compared to those of polyimide (PI)-based TENG, respectively. Then, we systematically investigate the effect of the rolling ball on the output performance of the devices. It is found that the output performance is increased with the enhancement of the radius of the rolling ball. Additionally, the solid sphere can also enhance its output performance compared to the hollow-sphere-based TENG, with the output voltage and output current reaching up to 32.2 V and 8.2 μA, respectively. Furthermore, we study the relationship between the output performance and the solid content. It is worth noting that there is an optimal concentration for the maximum output power. Furthermore, this TENG can harvest wave energy with various amplitudes and frequencies, which has the potential to effectively harvest random ocean wave energy for self-powered applications such as marine environmental monitoring.

## 2 Experimental Section

### 2.1 Fabrication of the Triboelectric Layer

The triboelectric materials used in this experiment were polymers such as polydimethylsiloxane (PDMS, Sylgard 184, Dow Corning), PI, and PTFE. First, the uncured PDMS (the base and curing agent were mixed at a weight ratio of 10:1) was poured on the Si substrate. After spinning at 2000 rpm for 15 s (spin coater, KW-4A), it was then cured at 90 °C for 1 h. The PI and PTFE used in this research were commercial smooth tapes with a thickness of 0.1 mm. The nano-PTFE solution (PTFE DISP 30) was purchased from Dupont. The initial density of the dispersion was at 60% solids, and the average particle size was about 220 nm. It was diluted to 45 and 30%, respectively, by deionized water in this experiment. The PTFE dispersion was span onto the PTFE film at 100 rpm for 10 s and then dried at 100 °C for 20 min, followed by 150 °C for 30 min.

### 2.2 Construction of a Rolling-Structured TENG

The triboelectric layer was sputter-coated with a layer of gold film using an Ion Sputter (SBC-12, China). The sputtering time was 90 s with a plasma current of 9 mA. The gold film with a thickness of 100 nm served as one electrode. Also, two polymer/Au films were attached to the inner surface of the waterproof ball with a distance of 1 cm. Before attaching the polymer/Au films, uncured PDMS was coated onto the inner surface. Then, the uncured PDMS was cured at 90 °C for 5 min. A small stainless ball was encapsulated into the waterproof ball with a diameter of 8 cm.

### 2.3 Characterization

The surface structure of PTFE was examined by scanning electron microscopy (SEM, FEI Quanta 450). The output voltage and output current were measured using an oscilloscope (MDO 3022) at a relative humidity of 50%. The sea wave was simulated by the designed wave pool powered by a pump.

## 3 Results and Discussion

**Figure 1:**
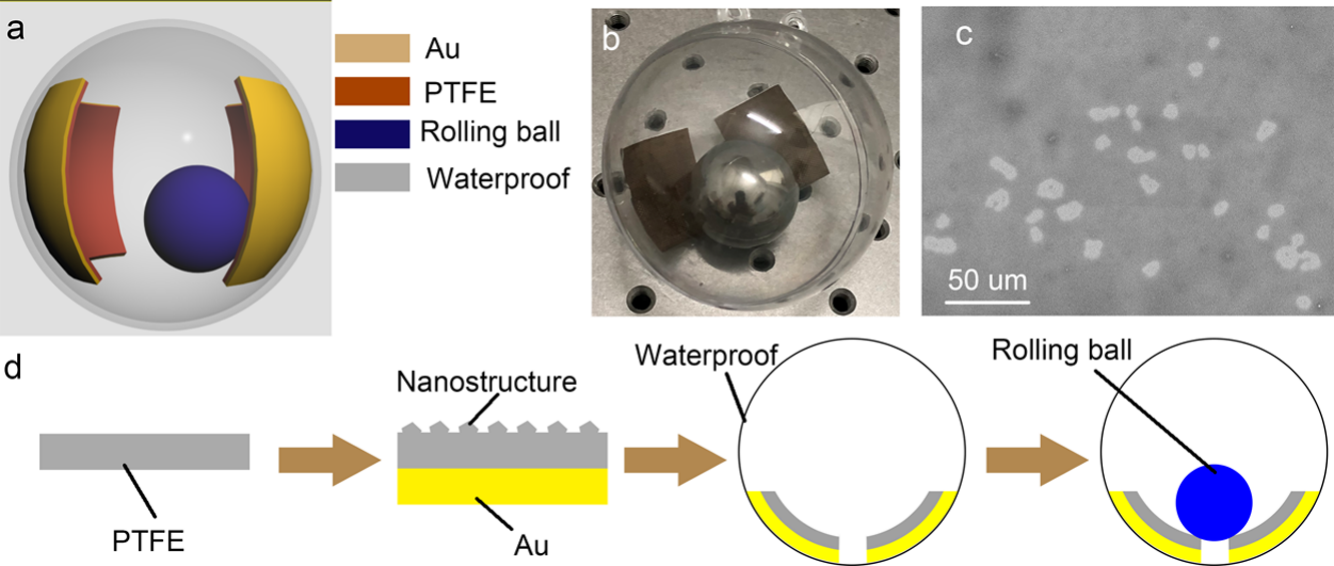
Device structure and the schematic fabrication process of TENG. (a) Device structure of the TENG. (b) Image of the proposed TENG. (c) SEM image of PTFE with a rough surface. (d) Fabrication process of the TENG.

The schematic for the fabrication process of TENG is illustrated in Figure 1. This device mainly consists of a movable part and two stationary films. The two films are made of a triboelectric layer and an electrode layer. As shown in Figure 1a, the small ball can be friction with the triboelectric layer under rolling movement. The image of the proposed TENG is displayed in Figure 1b. The diameters of the waterproof ball and the rolling ball are 8 and 3 cm, respectively. Each triboelectric layer is 2.5 cm in length and 2 cm in width with a gap distance of 1 cm. The stainless ball can roll freely inside the waterproof ball. The SEM image of PTFE is exhibited in Figure 1c. As can be seen, the nano–micro structure on the surface of the PTFE film can increase the effective contact area and enhance the friction effect, further increasing the generated triboelectric charges. Figure 1d schematically illustrates the fabrication process of TENG based on the rough PTFE film. The nano-PTFE solution is drop-cast onto the planar PTFE tape and then cured at 100 °C for 20 min, followed by 150 °C for 30 min. The nano–micro structure is created on top of the PTFE film to form a rough surface. Then, the Au electrode is sputter-coated on the back of the PTFE tape. Two PTFE/Au films are attached to the inner surface of the waterproof ball. The fabrication process and device structure are relatively simple.

**Figure 2:**
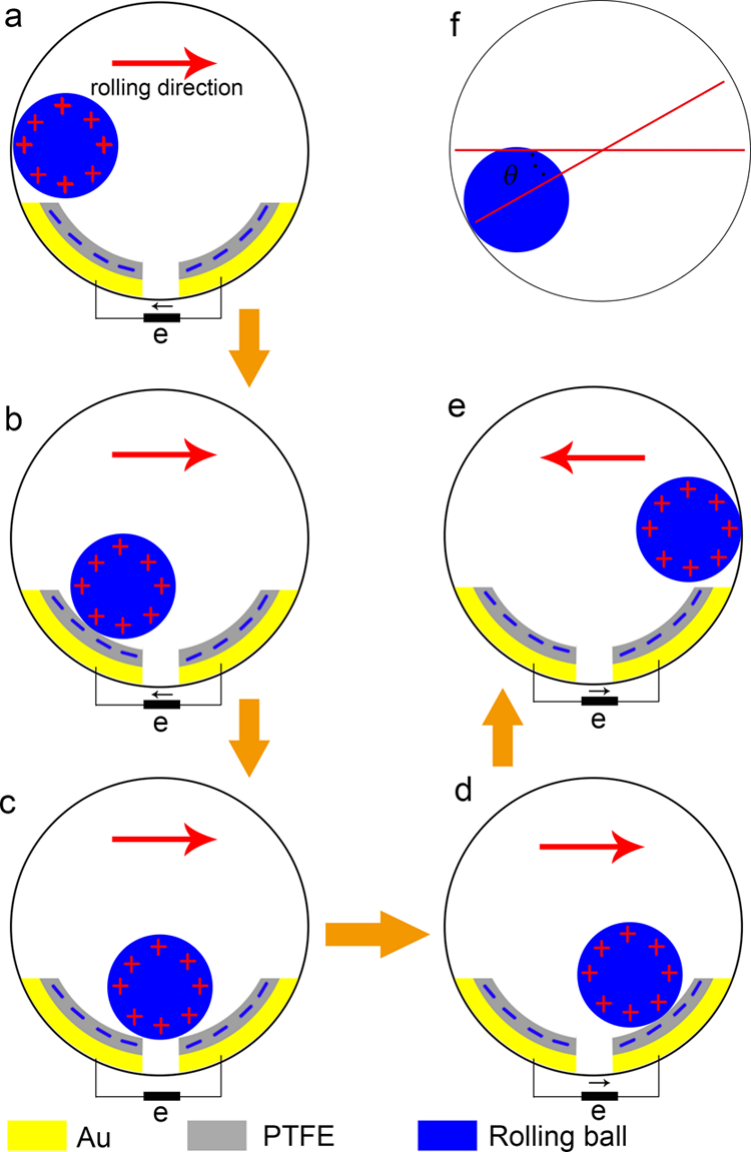
Illustrated operating mechanism of the rolling TENG. (a–e) Electron flowing direction in the rolling process. (f) Schematic angle of the rolling ball.

The operating mechanism of the TENG is depicted in Figure 2. After several rolling cycles, the stainless ball is positively charged and the two triboelectric layers are negatively charged. As shown in Figure 2a, in the initial state (θ = 0°), the rolling ball with positive charges and the triboelectric layer with negative charges cause the potential difference between the two electrodes under the triboelectric layers. As the ball rolled down (θ = 30°), the changes of the distance between the rolling ball and the two electrodes result in a potential difference between the two electrodes. This potential difference can drive the electron flow from the right electrode to the left electrode (Figure 2b). When the ball rolls to the bottom (θ = 90°), the potential difference between the two electrodes reaches 0 (Figure 2c). When the ball keeps rolling forward (θ = 120°), the changes of the potential difference between the two electrodes will result in a reverse direction of electron flow (Figure 2d). Finally, the potential difference between the two electrodes further decreases to the minimum value when θ = 180°(Figure 2e). It is worth noting that the positive charges distributed on the ball reach saturation after sufficient cycles.

**Figure 3:**
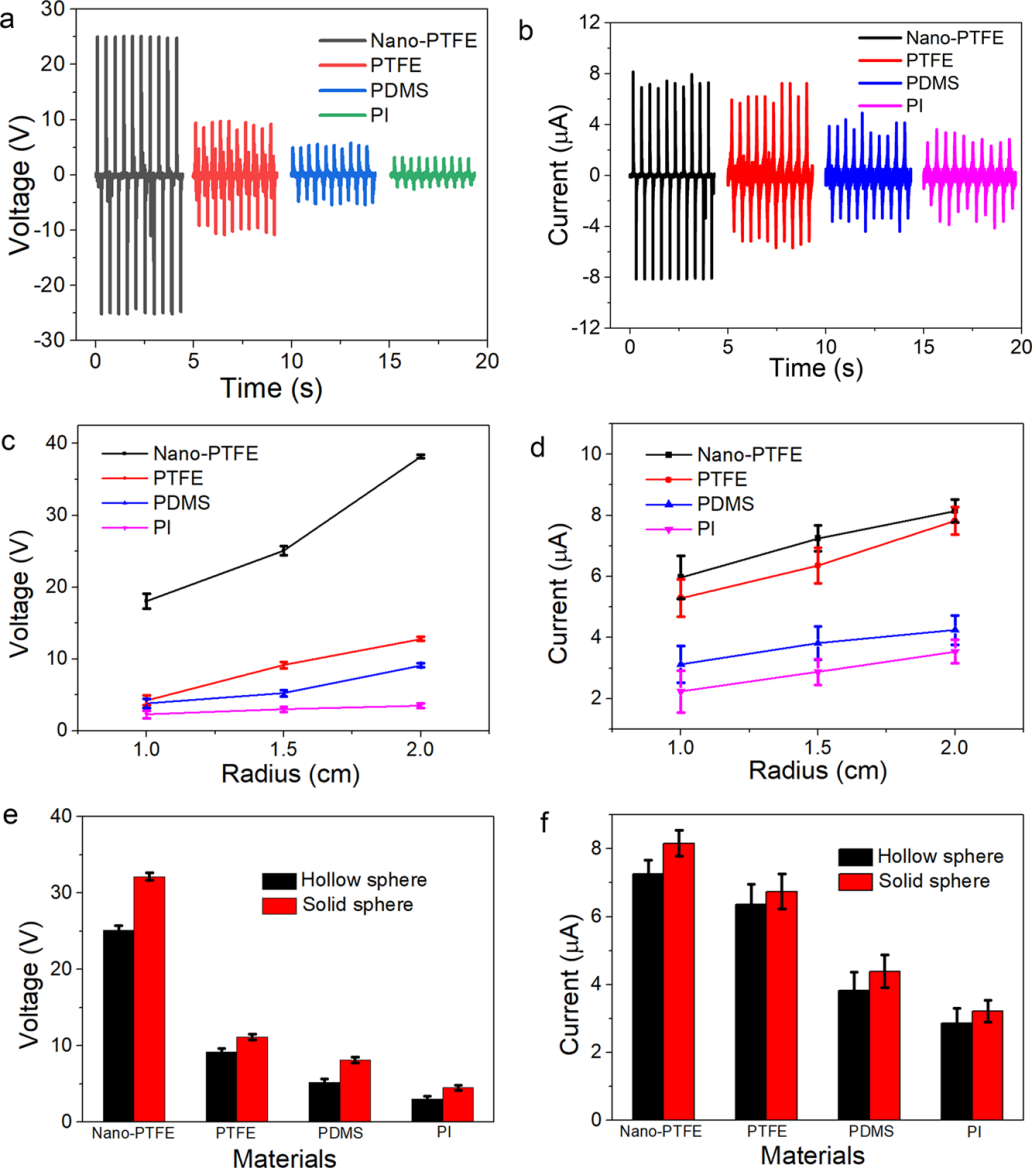
Comparison of output performances of various TENGs. (a) Output voltage of TENGs based on different triboelectric materials. (b) Output current of TENGs based on different triboelectric materials. (c) Relationship between the output voltage and the radius of the rolling ball. (d) Relationship between the output current and the radius of the rolling ball. (e) Compared output voltage of TENGs based on the hollow sphere and the solid sphere. (f) Compared output current of TENGs based on the hollow sphere and the solid sphere.

To investigate the output performance of the TENG, we fabricated a series of TENGs based on different triboelectric materials including nanostructured PTFE, PTFE, PDMS, and PI. In addition, the output performances of these TENGs based on various rolling balls are compared in Figure 3. At first, the comparisons of the output performances of TENGs which are based on various triboelectric materials are shown in Figure 3a,b. Hollow spheres with a radius of 1.5 cm are adopted in the control experiment. The devices are fixed on a testing platform driven by the swing motor with a frequency of 2 Hz, with a swing angle of about 80°. The output voltages of these TENGs based on nano–micro-structured PTFE, PTFE, PDMS, and PI are 25.1, 9.2, 5.2, and 3.0 V, respectively, while the output currents are 7.3, 6.4, 3.8, and 2.9 μA, respectively. The TENG based on PTFE shows higher output performance compared to the TENGs based on PDMS and PI, which is coincident with the triboelectric series.^39^ Furthermore, the output voltage and output current of TENG based on the rough PTFE film are increased about 2.7 times and 1.1 times compared to those of the PTFE-based TENG, which may result from the fact that the nanostructured surface can increase the effective contact area and enhance the friction effect.

Obviously, the parameters of the rolling ball, including the diameter and the weight, can directly affect the output performance. Therefore, the relationship between the output performance and the radius of the rolling ball is investigated, which is shown in Figure 3c,d. From the curves displayed in Figure 3c, it can be inferred that the output voltage is increased with the increase of rolling ball radius. For example, as the radius of the rolling ball increases from 1 cm to 2 cm, the output voltage of the nanostructured-PTFE-based TENG is increased from 18.1 to 38.2 V. The other three TENGs exhibit the same trend. This is due to the contact area increase with a larger radius, which results in more triboelectric charges. Hence, the output current is also increased as the radius increases. It is worth noting that the standard deviation of the output voltage and output current decreases with the increase of radius. This is attributed to the more stable movement of the rolling ball with a larger radius.

Then, the output performances of TENGs based on a solid sphere and a hollow sphere are compared at a frequency of 2 Hz with a swing angle of 80°. The results are exhibited in Figure 3e,f. Considering both the buoyancy of the waterproof material and the output performance, the rolling ball with a radius of 1.5 cm is chosen in this experiment. The TENGs based on solid spheres all show enhanced output performance. The solid sphere is heavier than the hollow sphere, which makes the rolling ball contact more tightly with the triboelectric layer during the movement. The full contact between the rolling ball and the triboelectric materials results in more triboelectric charges. This enhancement effect is more significant when the triboelectric layer has a rough surface. According to Figure 3, it can be concluded that the TENG exhibits higher output performance if a large solid sphere and nanostructured PTFE triboelectric material are used.

**Figure 4:**
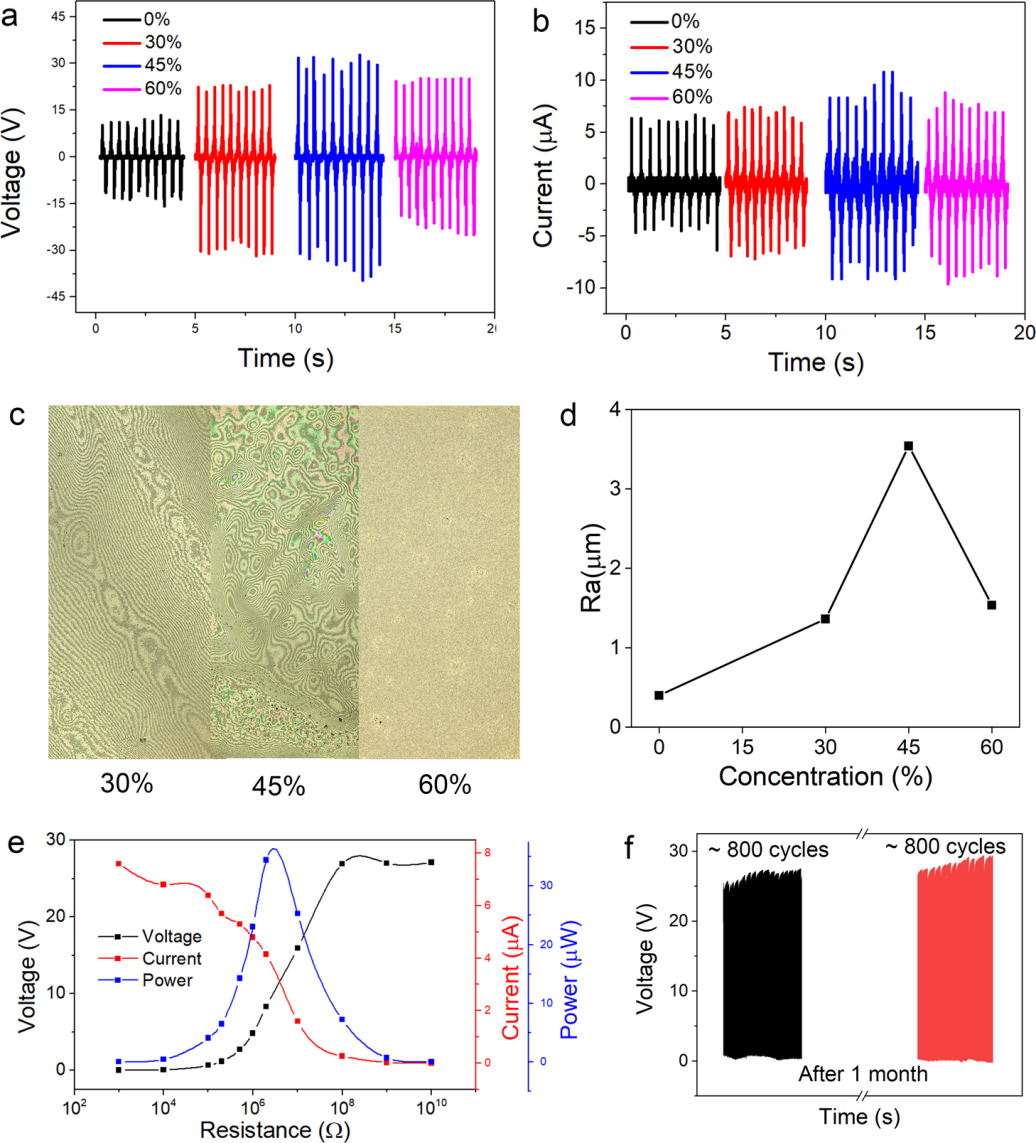
Output performance of the optimized TENGs. (a) Output voltages and (b) output currents of TENGs based on various nanostructured PTFE concentrations. (c) Surface morphologies of the PTFE films with different concentrations. (d) Relationship between the surface roughness and the PTFE concentration. (e) Relationship between the output performance and the load resistance. (f) Stability test of the TENGs.

Next, the output performance of nanostructure PTFE-based TENG is deeply investigated. A solid sphere with a radius of 1.5 cm is used in this study. The result is measured on the testing platform with a frequency of 2 Hz and a swing angle of 80°, and the output performance is shown in Figure 4. Figure 4a,b reveals the effect of the nanostructured PTFE concentration on the output voltage and output current. With the solid contents of the nanostructured PTFE being 0, 30, 45, and 60%, the corresponding output voltages are 11.5, 22.1, 31.7, and 24.8 V, respectively. The output voltage is increased as the concentration increases from 0 to 45%, which might result from the rough surface formed onto the PTFE tape. The rough surface enhances the effective contact area and thus the friction effect. However, with the increase of concentration, the nanostructured PTFE creates a relative planar film onto the PTFE tape, which will decrease the enhancement effect. The SEM images for the surface with different PTFE concentrations are displayed in Figure S1. It is obvious that PTFE with a 45% concentration shows a larger contact area than other concentrations. To quantitatively analyze the roughness, the surface roughness of the PTFE film is characterized using a 3D laser scanning microscope. The surface morphologies of the various PTFE films are shown in Figure 4c,d, which shows the surface roughness (*R*_a_). The *R*_a_ of the PTFE films with different concentrations are 0.39 μm (0%), 1.36 μm (30%), 3.54 μm (45%), and 1.55 μm (60%). The relationship between the output current and the concentration shows the same trend. In order to maximize the enhancement effect, the most appropriate concentration needs to be chosen. In addition, the relationship between the output performance and the load resistance is displayed in Figure 4e. The output voltage is increased with the load resistance, which becomes saturated at high resistance. Nevertheless, the output current shows the inverse trend. It is increased with the decrease of load resistance, and the maximum value is reached at low resistance. The instantaneous output power reaches the maximum value at a resistance of about 20 MΩ, with the maximum value being 34.5 μW. Then, the stability of the device is tested at a frequency of 1 Hz, with the test result presented in Figure 4f. As can be seen, the device is relatively stable after about 1000 times cycles. Also, the stability test is conducted again after 1 month. Moreover, the SEM images after the durability test in Figure S2 indicate that the nanostructure is relatively well preserved. The device shows great potential for long-term applications.

**Figure 5:**
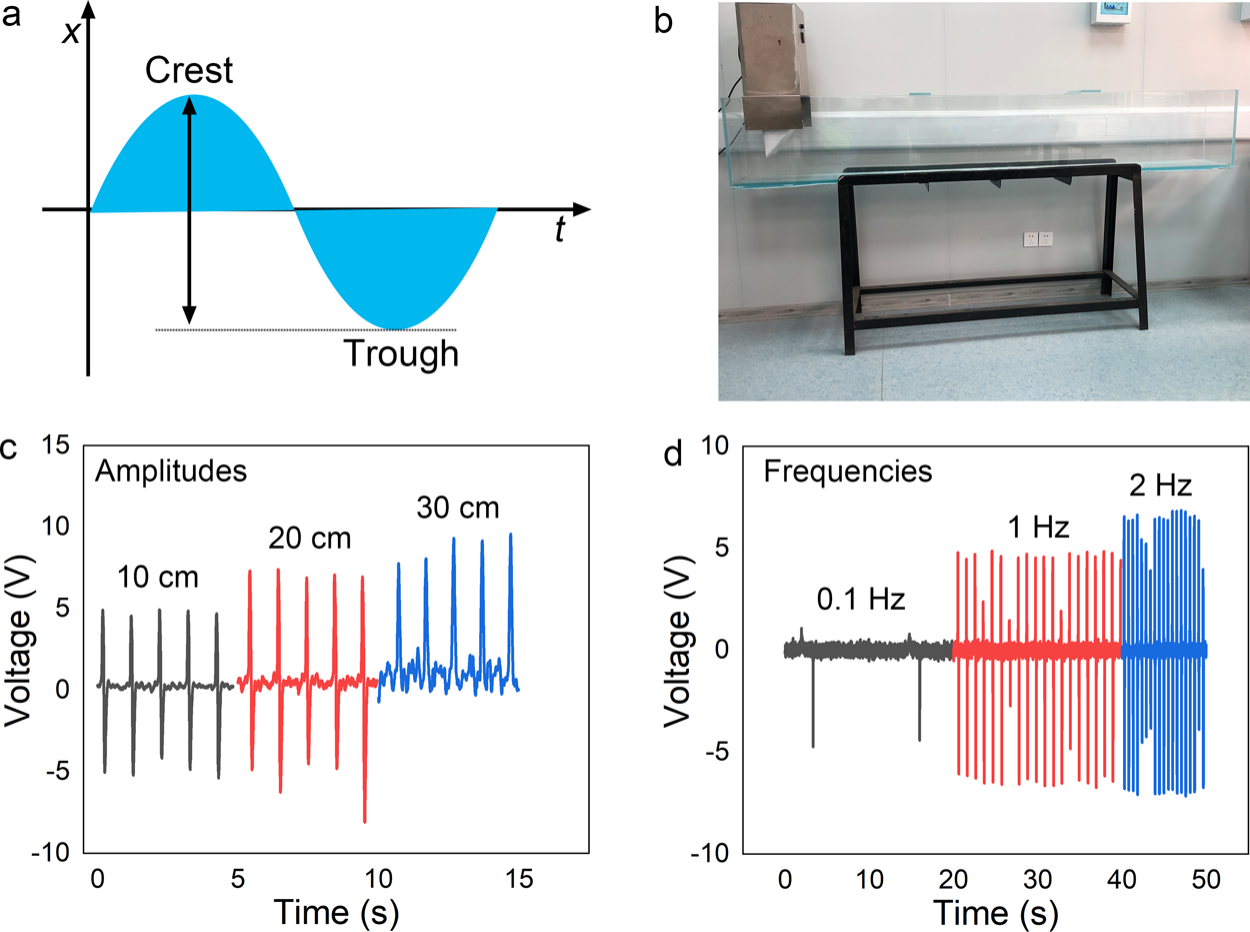
Applications of the environmental monitoring. (a) Schematic diagram of the wave. (b) Image of the designed wave pool. (c) Output voltages under different wave amplitudes. (d) Output voltages under different wave frequencies.

Finally, the applications of the TENG for environmental monitoring are demonstrated. Considering the buoyancy, the solid sphere with a diameter of 1.5 cm is adopted. The waterproof rolling TENG is placed in the designed wave pool (2.5 m in length and 0.5 m in width), which is used to control the wave amplitude and wave frequency. Also, the ball is fixed by a rope at the bottom of the pool. Figure 5a schematically illustrates the amplitude and frequency of the wave. The image of the designed wave pool is shown in Figure 5b. First, the relationship between the output voltage and the wave amplitude at a wave frequency of 1 Hz is presented in Figure 5c. The output voltages are 4.8, 7.1, and 8.5 V at the amplitudes of 10, 20, and 30 cm, respectively. In addition, the contact area between the rolling ball and triboelectric layer is increased with the amplitude. Thus, the average output voltage of TENG at a high amplitude is slightly increased. It should be noted that the ball may move irregularly at a high amplitude. Figure 5d compares the output voltage of TENGs at different wave frequencies. The typical water wave amplitude is 20 cm and the output voltage is slightly increased with water wave frequency, with more peaks at high frequency. Although the output performance is not uniform at simulated wave movement, it shows the potential to harvest ocean wave energy at various frequencies and amplitudes. Besides, the output voltage can be further increased by optimizing the structure parameters of the rolling ball and triboelectric layers.

## 4 Conclusions

In summary, a nano–micro PTFE-based rolling TENG with enhanced output performance is proposed for seawater energy harvesting. By introducing the nano–micro structure onto the planar PTFE surface, the effective contact area and the friction effect are significantly enhanced. Compared to the PI-based TENG, the output voltage and the output current are 25.1 V and 7.3 μA, which are increased by about 8 times and 2 times, respectively. Furthermore, the relationship between the nano-PTFE concentration and the output performance is analyzed in this paper. The TENG based on a 45% nano-PTFE concentration exhibits the maximum output performance. In addition, it is found that the output performance is increased with the enhancement of the rolling ball radius. The solid sphere can also improve its output performance. This TENG can harvest wave energy with various amplitudes and frequencies, which has the potential to effectively harvest random ocean wave energy for self-powered applications such as marine environmental monitoring.

## Supporting Information Available

Performance comparison of different TENG-based water wave harvesting technologies; SEM images with different concentrations of 30%, 45%, and 60%; and SEM images of PTFE before and after the durability test (PDF)
