# Supplementary material for: Optimization of a Rolling Triboelectric Nanogenerator Based on the Nano–Micro Structure for Ocean Environmental Monitoring
Source: ACS Omega. 2021 Aug 2;6(32):21059–65. doi: 10.1021/acsomega.1c02709 (PMC8375102; doi:10.1021/acsomega.1c02709)
Supplement: Supplementary file 1 — ao1c02709_si_001.pdf [file ao1c02709_si_001.pdf]

Supplementary Materials

Optimization of a rolling triboelectric nanogenerator based on nano-micro structure for ocean environmental monitoring

Huamin Chen[b], Jun Wang[b], Aifeng Ning[a,*]

[a] Donghai Institute of Ningbo University, Ningbo University, Ningbo Ciy, Zhejiang Province 315211, China;

[b] Fujian Key Laboratory of Functional Marine Sensing Materials, Center for Advanced Marine Materials and Smart Sensors, Minjiang University, Fuzhou City, Fujian Province 350108, China.

*** Corresponding author:** Aifeng Ning (ningaifeng@nbu.edu.cn)

Table S1. The performance comparison of different TENG based water wave harvesting technologies.

Year	Structure	Material	Power	Ref
Nov.2013	Water-involved	PDMS/Water	130 mW m^{-2}	S1
Jun. 2014	Water-involved	FEP/Water	0.12 mW	S2
Mar.2019	Water-involved	PTFE/Water	-	S3
Nov.2013	plane-structure	Kapton/Al	2.76 W m^{-2}	S4
Mar.2015	plane-structure	PTFE/Al	2.6 W m^{-2}	S5
Aug.2018	plane-structure	Al/FEP	15.97 mW	S6
Mar.2019	rolling spherical	Silicone rubber/Cu	45 mW	S7
Apr.2019	rolling spherical	Nylon/FEP	0.21 W m^{-2}	S8
Jun.2019	rolling spherical	FEP/Cu	32.6 W m^{-3}	S9
Apr.2017	bionic structure	Nylon/Kapton	1.366 W m^{-2}	S10
Oct.2019	bionic structure	PTFE/Cotton	-	S11

Dec.2019	bionic structure	Disk(Cu/Al)/PTFE	14.71 W m^{-3}	S12
Oct.2014	Hybrid structure	Water-involved+CS	41.2 +2.03 uW	S13
Apr.2019	Hybrid structure	FS+EMG	0.08+14.9 mW	S14
Sep.2019	Hybrid structure	SE+EMG	3.25 +79.9 W m^{-2}	S15

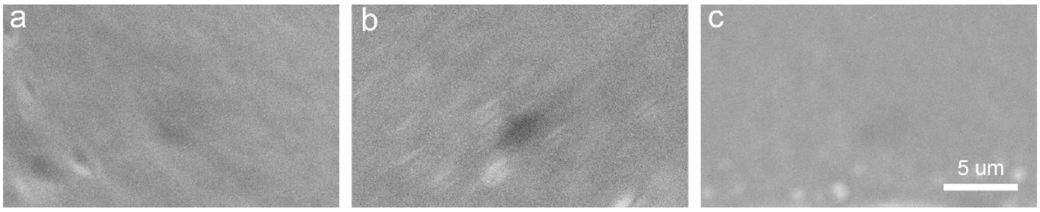

Figure S1. The SEM images with different concentrations of (a) 30%, (b) 45%, (c) 60%.

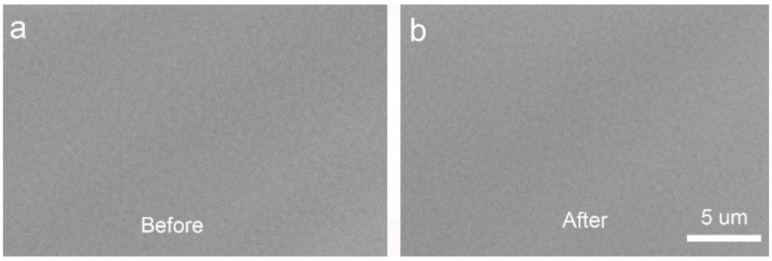

Figure S2. The SEM images of the PTFE before and after durability test. (a) The SEM image of the PTFE before durability test. (b) The SEM image of the PTFE after durability test.

Reference:

(S1) Lin, Z. H.; Cheng, G.; Lin, L.; Lee, S.; Wang, Z. L. Water-Solid Surface Contact Electrification and its Use for Harvesting Liquid-Wave Energy. *Angew. Chem.* **2013**, *125*, 12777-12781.

(S2) Zhu, G.; Su, Y.; Bai, P.; Chen, J.; Jing, Q.; Yang, W.; Wang, Z. L. Harvesting Water Wave Energy by Asymmetric Screening of Electrostatic Charges on a Nanostructured Hydrophobic Thin-Film Surface. *ACS Nano* **2014**, *8*, 6031-6037.

(S3) Xu, M.; Wang, S.; Zhang, S. L.; Ding, W.; Kien, P. T.; Wang, C.; Li, Z.; Pan, X.; Wang, Z. L. A highly-sensitive wave sensor based on liquid-solid interfacing triboelectric nanogenerator for smart marine equipment. *Nano Energy* **2019**, *57*, 574-580.

(S4) Hu, Y. F.; Yang, J.; Jing, Q. S.; Niu, S. M.; Wu, W. Z.; Wang, Z. L. Triboelectric Nanogenerator Built on Suspended 3D Spiral Structure as Vibration and Positioning Sensor and Wave Energy Harvester. *ACS Nano* **2013**, *7*, 10424-10432.

(S5) Chen, J.; Yang, J.; Li, Z. L.; Fan, X.; Zi, Y. L.; Jing, Q. S.; Guo, H. Y.; Wen, Z.; Pradel, K. C.; Niu, S. M.; Wang, Z. L. Networks of Triboelectric Nanogenerators for Harvesting Water Wave Energy: A Potential Approach toward Blue Energy. *ACS Nano* **2015**, *9*, 3324-3331.

(S6) Xiao, T. X.; Liang, X.; Jiang, T.; Xu, L.; Shao, J. J.; Nie, J. H.; Bai, Y.; Zhong, W.; Wang, Z. L. Spherical Triboelectric Nanogenerators Based on Spring-Assisted Multilayered Structure for Efficient Water Wave Energy Harvesting. *Adv. Funct. Mater.* **2018**, *28*, 1802634.

(S7) Cheng, P.; Guo, H. Y.; Wen, Z.; Zhang, C. L.; Yin, X.; Li, X. Y.; Liu, D.; Song, W. X.; Sun, X. H.; Wang, J.; Wang, Z. L. Largely enhanced triboelectric nanogenerator for efficient harvesting of water wave energy by soft contacted structure. *Nano Energy* **2019**, *57*, 432-439.

(S8) Liu, W. B.; Xu, L.; Bu, T. Z.; Yang, H.; Liu, G. X.; Li, W. J.; Pang, Y. K.; Hu, C. X.; Zhang, C.; Cheng, T. H. Torus structured triboelectric nanogenerator array for water wave energy harvesting. Nano Energy 2019, 58: 499-507.

(S9) Yang, X. D.; Xu, L.; Lin, P.; Zhong, W.; Bai, Y.; Luo, J. J.; Chen, J.; Wang, Z. L. Macroscopic self-assembly network of encapsulated high-performance triboelectric nanogenerators for water wave energy harvesting. *Nano Energy* **2019**, *60*, 404-412.

(S10) Ahmed, A.; Saadatnia, Z.; Hassan, I.; Zi, Y. L.; Xi, Y.; He, X.; Zu, J.; Wang, Z. L. Self-Powered Wireless Sensor Node Enabled by a Duck-Shaped Triboelectric Nanogenerator for Harvesting Water Wave Energy. *Adv. Energy Mater.* **2017**, *7*, 1601705.

(S11) Lin, Z.; Zhang, B.; Guo, H.; Wu, Z.; Zou, H.; Yang, J.; Wang, Z. L. Super-robust and frequency-multiplied triboelectric nanogenerator for efficient harvesting water and wind energy. *Nano Energy* **2019**, *64*, 103908.

(S12) Zhong, W.; Xu, L.; Wang, H.; Li, D.; Wang, Z. L. Stacked pendulum-structured triboelectric nanogenerators for effectively harvesting low-frequency water wave energy. *Nano Energy* **2019**, *66*, 104108.

(S13) Su, Y. J.; Wen, X. N.; Zhu, G.; Yang, J.; Chen, J.; Bai, P.; Wu, Z. M.; Jiang, Y. D.; Wang, Z. L. Hybrid triboelectric nanogenerator for harvesting water wave energy and as a self-powered distress signal emitter. *Nano Energy* **2014**, *9*, 186-195.

(S14) Hao, C. C.; He, J.; Zhai, C.; Jia, W.; Song, L. L.; Cho, J. D.; Chou, X. J.; Xue, C. Y. Two-dimensional triboelectric-electromagnetic hybrid nanogenerator for wave energy harvesting. *Nano Energy* **2019**, *58*, 147-157.

(S15) Hou, C.; Chen, T.; Li, Y. F.; Huang, M. J.; Shi, Q. F.; Liu, H. C.; Sun, L. N.; Lee, C. A rotational pendulum based electromagnetic/triboelectric hybrid-generator for ultra-low-frequency vibrations aiming at human motion and blue energy applications. *Nano Energy* **2019**, *63*, 103871.
